# Evaluation of immunotherapy efficacy in gynecologic cancer

**DOI:** 10.3389/fimmu.2023.1061761

**Published:** 2023-01-31

**Authors:** Genyi Jiang, Qianhua Wu, Bilan Li

**Affiliations:** ^1^ Department of Gynecology, Shanghai First Maternity and Infant Hospital, School of Medicine, Tongji University, Shanghai, China; ^2^ School of Medicine, Tongji University, Shanghai, China

**Keywords:** gynecologic cancer, immunotherapy, DNA mismatch repair (MMR), programmed cell death protein 1 (PD-1), tumor mutational burden (TMB)

## Abstract

Various immunotherapies have demonstrated remarkable success over the past few decades, and have been approved for the treatment of different cancer types. However, patient responses to immunotherapy are variable, and approximately 50% of cases are refractory to these agents. Tumor biomarker-based stratification of cases may therefore help identify subpopulations that are sensitive/resistant to immunotherapy; it may also improve prediction of response in various cancers including gynecologic cancer. These biomarkers include the tumor mutational burden, microsatellite instability, mismatch repair deficiency, T cell-inflamed gene expression profile, programmed cell death protein 1 ligand 1, tumor-infiltrating lymphocytes, and numerous other genomic alterations. Future directions in the treatment of gynecologic cancer include the utilization of these biomarkers to select ideal candidates. This review focused on recent advances in the predictive ability of molecular biomarkers in patients with gynecologic cancer who undergo immunotherapy. The most recent developments in combined immunotherapy and targeted therapy strategies and novel immune interventions against gynecologic cancers have also been discussed.

## 1 Introduction

Gynecologic cancer, mainly including endometrial cancer (EC), cervical cancer (CC), and ovarian cancer (OC), remains a leading cause of cancer-related mortality and represents a major challenge to women’s health. The prognosis for those with advanced and recurrent disease is dismal, with a low 5-year survival rate (1, 2). The treatments for gynecologic malignant tumors are increasingly diversifying, and include surgery, radiotherapy, chemotherapy, and immunotherapy. In this context, studies have recognized the role of comprehensive and multidisciplinary strategies for the radical treatment of tumors.

Owing to the development of molecular diagnostic technologies and improved understanding on the role of immune cell regulation in shaping the tumor microenvironment (TME), immunotherapeutic strategies developed in recent years have demonstrated remarkable clinical success in solid tumors. In particular, immune checkpoint inhibitors (ICIs) have revolutionized the treatment of cancer by providing durable remission; although the most promising results have been observed in metastatic melanoma and non-small cell lung cancer (3), numerous randomized controlled trials have also demonstrated their utility in gynecologic cancer (4–6). The targets for emerging ICIs include programmed cell death 1 (best known as PD-1) or its main ligand PD-L1, cytotoxic T lymphocyte-associated protein 4 (CTLA-4), and autologous T cells engineered to express a CD19-targeting chimeric antigen receptor (7). They enhance the function of effector T cells in antitumor responses, prevent T cell dysfunction and apoptosis, and antagonize immunosuppressive effects mediated by these immune checkpoints (8). Notably, approximately 20% of patients with most solid tumors respond to single-agent ICIs (9). Although ICIs have shown promising antitumor activity in gynecologic cancer, there are major challenges to their use. An inherent weakness of current treatment approaches for gynecologic cancer lies in the coexistence of therapeutic target pathways in both tumors and other tissues; this complicates the selection of appropriate treatment (8). The major biomarkers and immunotherapy efficacy differ between EC, CC, and OC (10). The different molecular types of each gynecologic tumor also determine the differences in immunotherapeutic benefits (11). Molecular subtype classification facilitates a more accurate characterization of tumor heterogeneity and provides insights into the prognostic and predictive relevance of immunotherapy (11). In this context, molecular typing of EC has been proposed for guiding appropriate use of immunotherapy (12) (**Table 1**). In view of these considerations, it is essential to summarize the potential biomarkers that may predict prognosis and immunotherapy efficacy.

**Table 1:** Four molecular subtypes of EC identified by The Cancer Genome Atlas (TCGA) /the Proactive Molecular Risk Classifier for Endometrial Cancer (ProMisE) (12).

Tumor types	POLE-ultramutated	mismatch repair deficient (MMRd)	TP53 mutant	No specific molecular profile (NSMP)
Previous naming	Polymerase Epsilon exonuclease domain mutated、	Microsatellite instability (MSI)	p53-mutated/Copy-number high	p53 wild-type/microsatellite stable(MSS)/Copy-number low
Somatic copy-number alterations (SCNA)	Very low	Low	High	Low
Mutational frequency	>100 mutations/Mb	100–10 mutations/Mb	<10 mutations/Mb	<10 mutations/Mb
Prognosis in early stage (I–II)	Favorable	Intermediate	Poor	Good/intermediate
Confirmatory test	Sanger/NGS Tumor mutation burden	MMR-IHC (MLH1, MSH2, MSH6, PMS2) MSI assay Tumor mutation burden	p53-IHC NGS Somatic copy-number aberrations	
Histological features	Endometrioid Grade 3 Significant TILs	Endometrioid Grade 3 Lymph vascular space invasion(LVSI) substantial MELF-type invasion Significant TILs lower uterine segment involvement	Serous Grade 3 LVSI	Endometrioid Grade 1–2 Squamous differentiation ER/PR(+)
Clinical features	EarlyStage (IA/IB) Early onset Young age	Correlated with Lynch Syndrome	Advanced stageLate onset	High BMI
Therapeutic method	Benefit from immunotherapy No significant difference in adjuvant treatment	benefit from immunotherapy	Adjuvant radiotherapy and chemotherapy	P13K/Akt pathway inhibitor

## 2 Theoretical basis of emerging immunotherapy

A deeper understanding on T cell-mediated antitumor immunity may lead to the development of new immune-based strategies offering durable clinical benefit. During presentation of a specific tumor antigen, the interaction between the major histocompatibility complex (MHC) and T-cell receptor provides the first signal for T-cell activation (13). However, co-stimulatory signals (such as those mediated by CD28 and CD80/86) are also required. Antigen processing and presentation enables the immune system to monitor cellular processes and precisely act upon cancer cells; this generates an effective antitumor immune response (14). Cancer immunotherapy leverages the cytotoxic potential of immune cells (especially tumor-specific cytotoxic T cells) in this process to control cancer progression (15). However, cancer cells escape elimination by cytotoxic T cells and suppress the effector function of tumor-specific T cells *via* numerous co-inhibitors (immune checkpoints such as PD-1, CTLA-4, lymphocyte activation gene 3, T cell immunoglobulin and mucin domain-containing protein 3, and T cell immunoreceptor with Ig and immunoreceptor tyrosine-based inhibitory motif domains). Notably, higher T cell immunoglobulin and mucin domain-containing protein 3 expression has been found to be associated with more advanced tumor stages in patients with OC (16). In most of the recent studies, T cell immunoglobulin and mucin domain-containing protein 3 and lymphocyte activation gene 3 were found to be co-expressed with PD-1 (17). In this context, PD-1 and CTLA-4 inhibitors have been studied extensively; they have shown dynamic and durable tumor regression in patients with EC, CC, and OC (18, 19). The production of ligands for checkpoint receptors can be a considerably effective immunosuppressive mechanism in gynecologic cancers. Emerging immunotherapy strategies block these immune checkpoints to disrupt negative regulation between tumor cells and T cells, which are considered to evoke antitumor immune responses. These agents are currently in clinical and preclinical stages of development (**Tables 2**, **3**).

**Table 2:** Clinical studies on the immunotherapy of gynecological tumors.

2.1. Endometrial cancer (EC)																
Tumor types		Therapeutic drugs of experimental group		Gov number		Type of study		Number of participants(n)		Biomarkers		ORR(%,95% CI)	Trial phase		PFS (months)	
EC		dostarlimab		NCT02715284 (20)		Single arm		104		dMMR		42.3%(30.6-54.6)	II		_	
EC		pembrolizumab		NCT02628067 (21)		Single arm		24		PD-L1,TMB		13%(2.8-33.6)	I		_	
Advanced EC		pembrolizumab		NCT02628067 (22)		Single arm		90		MSI-H/dMMR		48%(37-60)	II		_	
Solid tumors including EC		pembrolizumab		NCT02628067 (23)		Single arm; Cohort TMB-H and cohort Non-TMB-H		790		TMB-H(n=102) Non-TMB-H(n=688)		29%(21-39); 6%(5-8)	II		_	
Advanced EC		pembrolizumab plus lenvatinib		NCT03517449 (24)		Randomized controlled double-blind		827		dMMR		_	III		7.2 vs. 3.8, PFI-stratified HR:0.56 (95% CI, 0.47 -0.66; P<0.001).	
EC, CC		Pembrolizumab+radiation+immune/environmental-targeting compounds		NCT03192059 (25)		Single arm;3-cohort		EC:25 CC:18		–		–	II		–	
EC		pembrolizumab		NCT02899793 (26)		Single arm		Lynch-like cancers:25		TMB		100%(-)	II		–	
EC		dostarlimab		NCT02715284 (27)		Single arm. Cohort A1: dMMR/MSI-H and A2:pMMR/MSS		A1:n=129 A2:n=161		dMMR/MSI-H		A1:43.5%(34.0-53.4) A2:14.1%(9.1-20.6)	II		–	
**2.2 Cervical cancer (CC) and ovarian cancer (OC).**																
Tumor types		**Therapeutic drugs of experimental group**		**Gov number**		**Type of study**		**Number of participants(n)**		**Biomarkers**		**ORR(%,95% CI)**			**Trial phase**	
CC		dostarlimab		NCT02383212 (28)		Single arm		155		PD-L1		42.3%(30.6-54.6)			II	
CC		camrelizumab (anti-PD-1) +apatinib (anti-VEGF )		NCT03816553 (29)		Single arm		45		TMB-H		55.6%(40.0-70.4)			II	
OC		Pembrolizumab+ziv-aflibercept(anti-VEGF)		NCT02298959 (30)		Single arm		30		_		16.7%(7-32)			Ib	
epithelial OC		nivolumab +ipilimumab		NCT02498600 (31)		Randomized controlled double-blind		Experimental :51 Control :49		–		PFS(month):2 vs3,PFI-stratified HR: 0.53(95%CI,0.34-0.82)			II	
Refractory OC		intraperitoneal Olvi-Vec virotherapy		NCT02759588 (32)		Single arm		12		_		ORR:9%(-) Stable disease:64%			Ib	

**Table 3:** Ongoing clinical trials related to immunotherapy for EC in China.

Tumor types	Therapeutic drugs	Study registration number	Type of study	Number of participants	Trial phase	Treatment period
MSI-H/dMMR EC,squamous cell carcinoma of cervix,vulva	Pembrolizumab	CTR20200103	Single arm	1200	III	Second-line therapy
Advanced EC	Durvalumab + carboplatin, paclitaxel +Olaparide (PARP inhibitor)	CTR20210547	Randomized controlled double-blind	699	III	First-line/Initial therapy
Advanced EC	Pablizumab combined with carboplatin, paclitaxel+ radiotherapy	CTR20211275	Randomized controlled double-blind	990	III	First-line/Initial therapy
EC	Pabolizuma(anti-PD-1)+lenvatinib(anti-VEGF)	CTR20191858	Randomized controlled double-blind	875	III	First-line therapy
EC,cervical carcinoma,OC	PM8002 injection (Immunosuppression+anti-VEGF)	CTR20202497	Single arm	246	IIa	First-line therapy failure
Advanced EC	Sintilimab+fruquintinib(anti-VEGF)	CTR20190514	Single arm	323	Ib/II	First/second-line therapy failure
Advanced EC	IMP7068 (WEE1 inhibitor)	CTR20212068	Single arm	150	II/III	First/second-line therapy failure
Advanced EC	TQB2450 injection (anti-PD-1)+Anlotinib Hydrochloride(anti-VEGF)	CTR20213383	Single arm	196	I	First/second-line therapy failure
Advanced EC	KN035 injection(anti-PD-1)+lenvatinib(anti-VEGF)	CTR20212718	Single arm	108	II	First/second-line therapy failure

## 3 The status quo and efficacy prediction of immunotherapy

**Figure 1:**
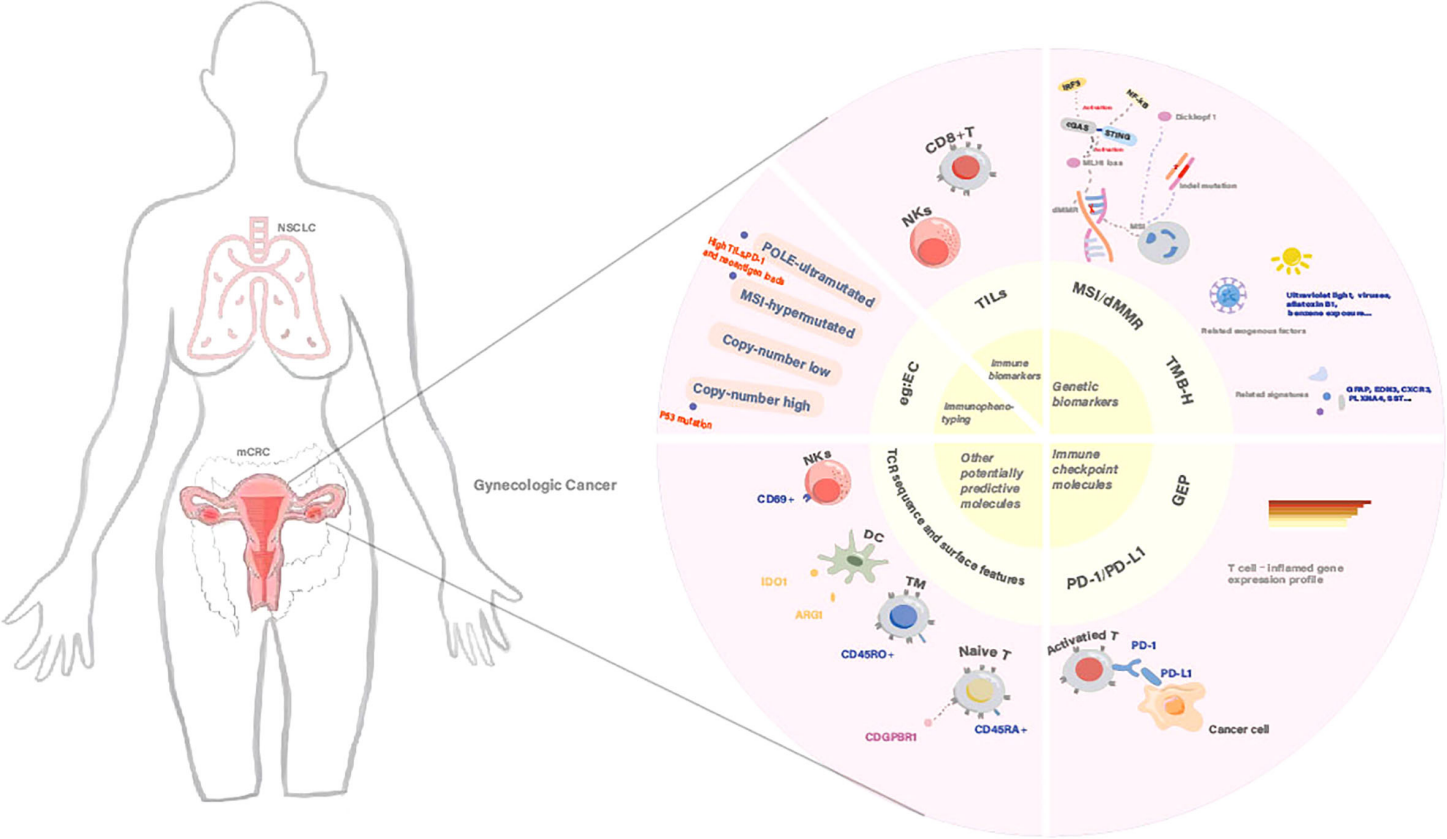
The status quo and efficacy prediction of immunotherapy in gynecologic cancers.

In current clinical guidelines, immunotherapy indications are based on the influence of tumor types, individual status, and genetic characteristics; these helps identify patients who are appropriate candidates for immunotherapy. As clinical application is based on the expected response to immunotherapy, the identification of sensitive/resistant subpopulations based on immune biomarkers is of particular significance. Extensive efforts are therefore being made to identify biomarkers with robust predictive values (**Figure 1**).

### 3.1 Genetic biomarkers and immune checkpoints

Emerging immune-relevant biomarkers for ICI-response may be broadly divided into two categories. One category is related to the tumor neoepitope burden, namely, genetic biomarkers such as the tumor mutational burden (TMB), microsatellite instability (MSI), and deficient mismatch repair (dMMR). The other category includes those with a T cell-inflamed TME (considering PD-L1 and T cell-inflamed gene expression profile as examples).

#### 3.1.1 MSI-high/dMMR

Cumulative evidence suggests that tumor neoepitope burdens are biomarkers that may predict the efficacy of ICIs. MSI-H/dMMR has demonstrated powerful efficiency in identifying patients whose tumors are sensitive to PD-1 and PD-L1 inhibitors. Notably, the human MMR system has remained conserved during evolution and involves a complex array of proteins. It is responsible for the recognition and repair of base mismatches and small-scale base deletions or insertions that occur during deoxyribonucleic acid (DNA) replication or recombination, particularly in stretches with repetitive DNA (such as microsatellites) (33). MMR proteins play an indispensable role in maintaining genome stability and the accuracy of genetic offspring. Mutations in the MMR gene ordinarily result in a hallmark phenotype known as MSI (34). MSI occurs due to changes in microsatellite sequence length, that are caused by insertion or deletion mutations during DNA replication. In this context, coding MSI is a mediator of dMMR tumor development (35). Gynecologic cancers such as EC and human papilloma virus-driven genital tract cancers with MSI have unique phenotypic features, such as an increased TMB and a higher number of tumor-infiltrating lymphocytes (TILs) (36). This may partly account for their superior response to ICIs.

MSI intensity is divided into four levels based on exact quantification of the detected genomic MSI level; these include the microsatellite stable, MSI-H, MSI-low, and the newly proposed MSI-intermediate levels (37). Germline mutations in human MMR genes have been confirmed to be genetic contributors to Lynch syndrome (LS); patients with LS therefore have an inherited predisposition for developing cancers with MSI. These mutations most commonly involve Mut S Homolog 2, Mut L Homolog 1 (MLH1), Mut S Homolog 6, and PMS1 Homolog 2; notably, MLH1 and Mut S Homolog 2 mutations account for most (90%) pathogenic mutations. In EC, the percentage of MSI ranges from 25% to 35%; approximately 5% are LS-related (approximately 17% of ECs with MSI) and have moderate prognosis (38). In this context, recent clinical studies have shown that treatment with pembrolizumab (a PD-1 inhibitor) confers better prognosis in LS-associated ECs than in sporadic MSI-H/dMMR ECs (39). It is believed that the high mutation burden of dMMR tumor cells leads to the presentation of considerable numbers of mutated neoantigens on MHC molecules to T cells, making these cancer cells highly recognizable by T cells. The TMB, characterized by sequence alterations in microsatellites, renders tumors immunogenic and sensitive to PD-1 inhibitors.

Despite tumor immunogenicity, patients with dMMR tumors experience highly variable responses and approximately half are refractory to treatment. However, the factors responsible for the variable response are largely unknown. In theoretical research, the proposed mechanisms mainly focus on immune escape and tumor growth (26). As the use of diagnostic strategies for accurate prediction of patient immune responses remains essential in clinical practice, both aspects aim to achieve a potent and durable antitumor response.

In EC, polymerase chain reaction-based tests for high MSI-H and dMMR generally yield highly concordant results. Although next-generation sequencing may help resolve discrepant MMR and MSI results (40), next-generation sequencing-identified MSI status and immunohistochemistry-identified MMR status are occasionally inconsistent in EC. However, MSI-H/dMMR determined by these two detection methods provides similar TMB and PD-L1 expression status (41). Notably, MSI-H and dMMR have been widely validated to be promising predictive markers of immunotherapy efficacy in EC. In this context, a phase I trial on intravenous dostarlimab in patients with EC showed more durable antitumor activity in the dMMR/MSI-H cohort (overall response rate: 43.5%) than in the MMR-proficient/microsatellite stable cohort (n=161, overall response rate: 14.1%); the agent demonstrated a manageable safety profile (27).

The MSI-H/dMMR-related signature MLH1 methylation status and the cyclic guanosine monophosphate-adenosine monophosphate synthase (cGAS)-stimulator of interferon genes (STING) pathway also have favorable predictive value. Gerlinger (42) suggested that the internal mechanism for this potentially predictive efficacy marker involves cGAS-STING pathway activation *via* MLH1 loss. Exo1 DNA exonuclease is poorly controlled in cases of MLH1 loss; this leads to chromosome instability, escape of nuclear DNA into the cytosol, and consequent activation of the cGAS-STING pathway. Thus, as confirmed by studies in several clinical cohorts and mouse models, individuals with high cGAS-STING expression have better and longer responses to ICIs; as the critical promoter of immune recognition, cGAS-STING may therefore be a prerequisite predictive biomarker for immunotherapy efficacy. Lu et al. (43) drew a similar conclusion and suggested that dMLH1 tumor cells accumulate cytosolic DNA and produce interferon (IFN)-b *via* the cGAS-STING-dependent pathway; this renders dMLH1 tumors highly sensitive to ICIs. In this context, the analysis of data from humanized mouse models with defective MLH1 (dMLH1) and clinical trial samples have revealed other mechanisms of ICI-responsiveness in dMMR cancers in addition to neoantigen expression. The findings suggest that dMLH1-triggered cell-intrinsic DNA sensing in tumors can enhance cross-priming of CD8+ T cells by dendritic cells; this is achieved *via* activation of the cGAS-STING pathway. The findings also suggest that a deficiency of cGAS-STING pathway components in dMMR tumor cells considerably reduces tumor infiltration by CD8^+^ T cells; as this does not necessarily involve mutation-mediated neoantigens, it demonstrates the conclusions in reverse order. In their study, Ghosh et al. found that mutant P53 (a tumor suppressor gene) suppresses innate immune signaling by altering signaling *via* the cGAS-STING pathway, interfering with its downstream signal to TBK1, and inhibiting IRF3-induced apoptosis; this prevents phosphorylation of the substrate and suppresses immune surveillance (44). In EC, findings from clinical trials also suggest that the MLH1 methylation status predicts response to adjuvant therapy (45). Another dMMR/MSI-related signature, namely, Dickkopf 1, has been reported to suppress the antitumor immune response *via* the glycogen synthase kinase-3β/transcription factor E2F1/T-bet axis in CD8^+^ T cells; this predicts poor responses to PD-1 inhibitors in patients with dMMR/MSI colorectal cancer (CRC) (21).

#### 3.1.2 TMB

The TMB is defined as the total number of mutations present in the tumor specimen; this reflects the quantity of mutations in the cancer. A higher TMB reflects a higher number of mutations, which may be processed into a larger number of neoantigens, increasing the opportunity for T cell recognition; this is related to better clinically efficacy of ICIs. A TMB-high (TMB-H) condition is defined by >10 mutations/Mb of DNA. In 2020, the United States Food and Drug Administration (FDA) approved whole-exome sequencing as an auxiliary diagnostic approach in patients with TMB-H tumors receiving pembrolizumab (a monoclonal antibody targeting PD-1), regardless of histology (22). Another method for the assessment of TMB involves evaluation of circulating tumor DNA levels by liquid biopsy. A TMB-H status has recently been found to have considerable predictive value for clinical responses to ICI therapy in patients with MSI-H and even microsatellite stable tumors. A higher TMB is prognostic of a better prognosis, regardless of the status of other biomarkers and types of treatment. It is therefore an important factor in EC. The level of TMB and the neoantigen immunogenicity it processes are related to genomic signatures created by exogenous mutagens such as ultraviolet light, smoking, viruses, aflatoxin B1, and benzene exposure; however, this is not universally observed across various tumors. In recent years, positive results have been most obtained in studies on melanoma, lung cancer, urothelial carcinoma, and squamous cell carcinoma of the skin (where the highest clinical benefit has been obtained) (46).

Several immune therapies have been currently approved for TMB-H gynecologic cancers. The multi-cohort open-label nonrandomized phase 2 KEYNOTE-158 study included 1073 patients from 81 academic facilities across 21 countries; they were treated with pembrolizumab for advanced solid tumors (eligible tumor types included CC and EC, among others) and the association between antitumor activity and TMB status was assessed. Objective responses were observed in 30 of 102 (29%; 95% confidence interval: 21-39) and 43 of 688 (6%; 5-8) patients in the TMB-H and non-TMB-H groups, respectively. Notably, the TMB is a novel and useful predictive biomarker for robust responses to pembrolizumab monotherapy in patients with previously treated recurrent or metastatic solid tumors (47). In a phase II trial on camrelizumab (PD-1 inhibitor) plus apatinib (vascular endothelial growth factor [VEGF] receptor-2 inhibitor) in patients with advanced CC, Huang et al. found that TMB-H was associated with longer progression-free survival (PFS) (hazard ratio: 0.26, p<0.01) and overall survival (OS) (hazard ratio: 0.31, p=0.05); it could therefore also be a novel predictive biomarker in patients with CC who are treated with PD-1 inhibitor-based combination therapy. The study also found that the TMB-related signature (GFAP, EDN3, CXCR3, PLXNA4, and SST) meaningfully predicted OS in EC (23). In this context, although OC is expected to have a high TMB owing to deficient DNA repair, it is considered to be a “cold tumor” with a TMB-low phenotype (median TMB: 3.6 Mb). Notably, TMB has not consistently demonstrated a positive predictive effect in studies on OC (18).

#### 3.1.3 Other genomic alterations

Switch/sucrose non-fermentable (SWI/SNF) complexes are specialized protein machinery complexes, which are able to restructure the nucleosome (48). The cancer-promoting role of mutations in SWI/SNF genes has only been recognized recently; they are associated with responsiveness to ICIs. This study focuses on genomic alterations related to immunotherapy of gynecological tumors.

The AT-rich interaction domain 1A (ARID1A) is a subunit of the chromatin remodeling complex SWI/SNF; it facilitates access of proteins to DNA (49). ARID1A alterations compromise the MMR pathway and increase the number of TILs and PD-L1 expression in some cancers (50). Thus, ARID1A mutations are not only considered as a prognostic biomarker, but also as a potential predictor for the efficacy of ICI therapy; they are also considered as a target for therapeutic interventions (51). Certain studies (52) have suggested that cancers (especially EC) with multiple ARID1A alterations have higher TMB and markedly high immune infiltration levels; this indicates the value of ARID1A alterations as a predictive biomarker for response to ICI treatment.

Other genomic alterations related to SWI/SNF complexes, which are rarely found in gynecological cancers, have also been proposed to have predictive value for immunotherapy in malignant tumors; these include polybromo-1, SMARCB1, and SMARCA4 loss, among others (53, 54).

In this context, Liao et al. (53) found APOBEC3B to be consistently enriched in patients with gastric-type cervical adenocarcinoma having a favorable prognosis; this suggests that recurrent APOBEC3B alterations have potential prognostic value in the immunotherapy of gynecological cancers.

Protein kinase, DNA-activated, catalytic polypeptide (PRKDC) encodes the DNA-dependent protein kinase catalytic subunit protein, which plays an important role in nonhomologous end joining of DNA double-strand breaks (55). Loss of PRKDC expression is associated with impaired DNA repair (56). Studies on CC and EC have shown that PRKDC mutations are significantly associated with a high TMB and MSI-H status. In a study on the CT26 animal model, PRKDC knockout or DNA-PK inhibition was found to enhance the efficacy of anti-PD-1 therapy (57). Therefore, PRKDC may not only be a predictive biomarker, but also a drug target for ICIs.

#### 3.1.4 PD-1/PD-L1

PD-L1 expression is one of the most widely studied factors for sensitivity to ICI treatment. The FDA has approved immunohistochemically assessed cell membrane PD-L1 levels as a predictor of treatment effectiveness; this is used in several cancers such as melanoma, gastrointestinal tumors, non-small cell lung cancer, and EC. However, it is only considered to have predictive value when evaluated before treatment. In this context, PD-1 expression also has prognostic value. In their study using data from The Cancer Genome Atlas database (n = 356) and the Fudan University Shanghai Cancer Center cohort (n = 276), Li et al. (58) found that higher PD-1 and PD-L1 expression correlated with better prognosis in patients with CRC. In the case of CC, patient selection based on PD-L1 protein expression showed low response rates. In the study by Rotman et al. (59), testing of PD-L1 and PD-L2 expression using fluorescence *in situ* hybridization and immunohistochemistry was not found to be optimal, as PD-L1 and PD-L2 were associated with interferon induction and not gene amplification. The authors found a strong correlation between PD-L1/L2 and INF-γ expression/transcript levels; they therefore suggested that ribonucleic acid (RNA) *in-situ* hybridization, in conjunction with IFN signaling evaluation, is a more promising technique for immune checkpoint detection in CC. In this context, a phase I clinical trial (60) analyzed 155 samples of CC to explore the potential relationship between PD-L1 expression and histology; the findings suggested that both immune cell presence and PD-L1 expression in tumor cells were more common in squamous cell carcinomas than adenocarcinomas. The findings suggested that cemiplimab (a PD-1 inhibitor) has activity in squamous cell CC. Notably, a clinical trial from China found that high-grade epithelial OC (EOC) demonstrated significantly higher expression of both PD-1 and PD-L1 than low-grade EOC; on Kaplan-Meier survival analysis, high PD-1 and PD-L1 expression was an indicator of poor prognosis in these patients (28). However, there are still limitations to the use of PD-L1 expression levels for evaluating responses to immune checkpoint blockade; these include the lack of standardization for both, detection methods for the heterogeneous expression of PD-L1 in the tumor microenvironment and definition of PD-L1 positivity (61).

#### 3.1.5 Gene expression profile

The gene expression profile is another emerging predictive biomarker for response to pembrolizumab; similar to PD-L1, it is an inflammatory biomarker indicative of a T cell-inflamed TME. In their study on 475 samples from patients with advanced solid tumors (including 22 types of solid tumors), Ott et al. (62) explored the combined potential of the gene expression profile and TMB in predicting the clinical response to PD-1 inhibitors. They assessed four cohorts separately after performing rigorous stepwise tests using databases such as The Cancer Genome Atlas. The results showed that these two emerging predictors are independent and only show moderate correlation; they may therefore be used in conjunction to determine the potential target biological model related to their respective groups.

### 3.2 Immune biomarkers: TILs

Studies have shown that pretreatment TIL clonality is predictive of ICI benefits in solid tumors. TILs are of paramount importance for effective antitumor immune responses. They include a heterogeneous group of lymphocytes, including effector T cells, regulatory T cells, functionally exhausted T cells, natural killer cells, macrophages, dendritic cells, myeloid-derived suppressor cells, and other immune cells (63). A trial (64) found that the T cell receptor repertoire of TILs may be indicative of responses to immunotherapy. The findings also suggested TIL diversity to be prognostic for OS across several cancers in the absence of anti-PD-1 inhibitor therapy. Notably, ICIs have limited efficacy in high-grade serous OC. In this context, a study (65) had utilized 12 patient-derived high-grade serous OC organoid co-cultures to detect key cellular and mechanistic targets evading current therapies. Although natural killer cells are a key missing component in the current understanding on ICI-induced immune response in high-grade serous OC, the findings demonstrated that state changes in both natural killer cells and a subset of CD8+ T cells are critical in achieving effective antitumor immune responses in this cancer. The findings therefore suggest that immune therapies (such as BRD1 inhibitors) that induce such cellular state changes can improve the limited efficacy in these tumors.

### 3.3 Immunophenotyping

Findings from clinical studies have validated the improvement in prognostic accuracy offered by the integration of molecular classification with conventional clinicopathological findings in intermediate-risk patients with advanced tumors. Various tumors, including gynecologic tumors, have currently been standardized by immunophenotyping multiparameter flow cytometry. The Cancer Genome Atlas cohort was divided into 4 subgroups (by molecular features), namely, “POLE-ultramutated”, “microsatellite instability (MSI)-hypermutated”, “copy-number low”, and “copy-number high”. The subgroups showed distinct prognostic differences, having a significant impact on the treatment of EC. The first two groups were characterized by high neoantigen loads, more TILs, and overexpression of PD-1 and PD-L1, all of which predicted a better treatment outcome. The last group was characterized by abnormal P53 expression and had the worst prognosis. Studies across various cancer types have suggested that POLE is related to TMB, while P53 mutations are not significantly associated with TMB (66).

### 3.4 The cyclooxygenase 2-prostaglandin E2-prostaglandin E2 receptors axis

The signaling of cyclooxygenase 2 (COX-2)-prostaglandin E2 (PGE2)-PGE2 receptors is considered to be the central inflammatory pathway involved in gynecologic carcinogenesis (67), including angiogenesis, tumor cell proliferation, migration, invasiveness, apoptosis, inhibition, and immune evasion (68). COX-2 is a rate-limiting enzyme responsible for the conversion of arachidonic acid to prostaglandins (69). High levels of PGE2 can suppress IFN-γproduction, antigen presentation, and inhibit CTL proliferation and activation (70).

Numerous studies in gynecological malignancies, including EC (71), EOC (72, 73), and CC (74), have demonstrated the expression of the whole signaling pathway to be upregulated. In addition, extensive research has confirmed COX-2 to be a negative predictor of disease relapse in patients with EC (73, 75). COX-2 may therefore represent a novel and specific anti-inflammatory target for immunotherapy in gynecologic cancer. Studies are also providing newer evidence for combining selective COX-2 inhibitors with immunotherapy (67). Other downstream targets in this axis are also being widely studied to identify more promising specific biomarkers for immunotherapy in gynecologic tumors; these include: angiogenic factors (VEGF) (76), anti-apoptotic factors (Bcal-2) (76), and chemokines (77) (MIP-1α and MIP-1β) and their receptors or mediators.

### 3.5 Other potentially predictive molecules

Recent studies suggest that the overexpression of a bile acid receptor, namely, G-protein-coupled bile acid receptor-1, in SOC promotes proliferation and predicts a poor prognosis; it may better classify these tumors based on the molecular profile, and is a potential drug target (78). Other mutation types include missense mutations (novel nonsynonymous single-nucleotide variations) and insertions/deletions. In their study, Mandal et al. found that the resultant mutational load partly underlies the variable response to PD-1 blockade immunotherapy in dMMR tumors (including EC); they also found that the extent of response is particularly associated with the accumulation of insertions/deletions and rarely associated with single-nucleotide variations mutations (37). Numerous preclinical studies on metastatic cancers have shown that accelerated tumor growth and enhanced resistance to immunotherapy are related to increased levels of indoleamine 2,3-dioxygenase 1 and arginase 1 in tumor-infiltrating dendritic cells and many other immunosuppressive enzymes/surface molecules produced by tumor-infiltrating cells (including IL-10, coinhibitory receptors, and other failure markers). In both preclinical and clinical studies, a diversified T cell receptor repertoire and surface features of an active multiepitope memory T cell response (such as Ki67 positivity, CD45RO-to-CD45RA switching, and CD69 expression switching) have been found to be related to improved responses to immunotherapy (79).

Glycodelin is a secreted glycoprotein expressed in reproductive tissues such as the endometrium, decidua, seminal plasma, and amniotic fluid (76). Certain studies (80) suggest that over-expression of glycodelin plays a role in carcinogenesis; they have identified it as an important biomarker for immunomodulatory functions in EC. However, further studies are warranted as data regarding its practical use in predicting immunotherapy efficacy are lacking.

## 4 Current clinical application of immunotherapy in gynecologic cancer

### 4.1 ICIs

The FDA approved monotherapy with pembrolizumab and dostarlimab (TSR-042) in 2017 and 2021, respectively (81), for use in adult patients with recurrent or advanced dMMR/MSI-H EC that has progressed on or after platinum-based chemotherapy. These two anti-PD-1 antibodies have performed well in multiple trials. For instance, in the GARNET trial (82), which evaluated the response to intravenous dostarlimab in dMMR EC patients from 7 countries, the tumors had shrunk successfully in 42% (n=104) of patients; the agent also showed clinically durable antitumor activity with an acceptable safety profile in patients with dMMR ECs who had received prior platinum-based chemotherapy. In their report on the nonrandomized open-label multi-cohort phase II KEYNOTE-158 study (NCT02628067), O’Malley et al. (21) indicated that pembrolizumab demonstrated robust and durable antitumor activity in patients with EC, with controllable adverse effects.

Recent phase I and III studies on CC have shown encouraging results. Patients with recurrent CC after first-line platinum-containing chemotherapy experience significantly longer survival with cemiplimab than with single-agent chemotherapy (20, 60).

However, there are still no approved immunotherapy agents for OC. Several antibodies directed against PD-1, PD-L1, and CTLA-4 are being tested clinically. Although the response rates are considerably modest, published data support the use of ICIs as a potentially valuable therapeutic approach in OC (65). However, studies are more likely to employ combined therapies than immunotherapy alone (6).

### 4.2 Immunotherapy combined with other tumor-targeted therapies

Anti-PD-1 and PD-L1 trials have significantly shifted focus from single immunotherapy agents to combination strategies. Since anti-PD-1 and PD-L1 monoclonal antibodies were approved by the FDA for standardized clinical trials (from 2018 to December 2019), combined trials have comprised 1716 (76%) of 2250 trials worldwide (83).

In this context, the combination of ICIs with targeted chemotherapeutic drugs has shown potential efficacy in EC. To illustrate, VEGF is one of the most important angiogenic factors that promote the development and progression of tumors by increasing microvascular permeability and directly stimulating endothelial cell growth and angiogenesis; a recent clinical study (84) on 173 patients with EC found that the serum levels of VEGF and components of the plasminogen activator system at primary diagnosis correlated significantly with the prognosis and clinicopathological risk factors, including disease stage, tumor histology, tumor grade, myometrial invasion, and lymph node metastases. In particular, high concentrations of plasminogen activator inhibitor-1 and -2 and tissue plasminogen activator were found to be independent factors for poor prognosis. Anti-VEGF drugs, including bevacizumab and lenvatinib (multi-targeted tyrosine kinase inhibitors of VEGF), inhibit tumor development by inhibiting tumor angiogenesis. Anti-VEGF treatment results in decreased expression of CXCL8, which is a mediator of the inflammatory response and angiogenesis, among other processes. Anti-VEGF therapy additionally increases the expression of B cell and natural killer cell genes, as well as genes related to innate cell chemotaxis or the complement cascade.

In this context, anti-epidermal growth factor receptor treatment is believed to increase the expression of immune components in the TME in addition to antitumor effects (85). Clinical studies in EC also show a positive correlation between mutant p53 and VEGF expression. Based on these findings, these targeted drugs are currently used as second-line treatment for advanced/recurrent EC. In the context of combination with immunotherapy, the viewpoint 309/KEYNOTE-775 study showed that lenvatinib plus pembrolizumab significantly increased PFS and OS in patients with advanced EC. This combination is worth using, as several recent clinical trials have comprehensively evaluated its curative effect and risks. In their phase III randomized controlled trial (with a total of 827 samples in a 1:1 ratio) (4), Makker et al. found the degree of adverse reactions to be mostly limited to grades 1 and 2; they were mostly controlled in time by dose reduction, treatment interruption, or withdrawal. Clinical trials on OC have drawn similar conclusions (24). However, the scope of these trials was limited by the inadequate duration of follow-up (approximately 10 months) and possible missing data on long-term adverse reactions. In their review, Liu et al. (30) included studies on multiple animal models, which supported the notion that p53-dependent apoptosis induced by chemotherapeutic drugs (predominantly DNA damaging agents) may reverse the resistance to immunotherapy and sensitize tumors that are unresponsive to ICI therapy.

MLN924 (a selective NEDD8-activating enzyme) was recently found to preferentially target dMMR cells and change the misfolding of proteins; the accumulation of mutant proteins was identified as a novel treatment for MSI tumors, where proteome instability represents a target with therapeutic vulnerability. In their study, McGrail et al. (86) found that MLN924 induced immunogenic cell death in MSI tumors; combination with immunotherapy enhanced efficacy and could even reverse failure to PD-1 inhibitor therapy, thereby maximizing the number of patients with a clinically meaningful response. The authors validated their findings by establishing isogenic endometrial and CRC tumor model cell lines in mice.

A clinical trial that included 100 patients who were treated with a combination of nivolumab and ipilimumab (87) found that the combination offered a superior response rate and longer PFS compared with nivolumab alone, with similar toxicity as that of previous reports. In another study, the combination of pembrolizumab and pegylated liposomal doxorubicin showed preliminary evidence of clinical benefit (31). These findings suggest that although there are currently no approved immune therapies for OC, combination therapy shows superiority (albeit limited) over immunotherapy alone.

However, compared with standard chemotherapy, combined treatments were associated an increase in adverse events (AEs) (both in degree and scope); common ICI-related AEs included fatigue, diarrhea, pruritus, thyroid dysfunction, hepatitis, arthralgia, fever, rash, pruritus, and endocrinopathies such as thyroiditis and pancreatitis. Low-grade endocrine disorders, and mainly hypothyroidism, are among the common AEs. The wide range of AEs associated with combined treatment may be severe, making its clinical use controversial. To illustrate, in a study on combined treatment with nivolumab and ipilimumab, 13% patients had adverse reactions; autoimmune diseases and acute childhood injury were particularly common (25).

### 4.3 Other immunotherapies under extensive investigation

Tumor-specific antigens produced by spontaneous mutations in tumors have the potential to predict the susceptibility to tumor vaccine therapy. Trials have been conducted using different antigen presentation methods to develop vaccines based on molecules (peptide, DNA, and RNA), cells (tumor cells and dendritic cells), and carriers (viruses, bacteria, and yeasts). In a phase I/IIa clinical trial on MMR-deficient cancers, Kloor et al. (88) demonstrated the promise of a novel approach using a frameshift peptide neoantigen-based vaccine.

Bioinformatics algorithms and high-throughput sequencing are generally used to predict the combination of peptides and MHC proteins (9). Oncolytic peptides are a class of anticancer agents derived from natural antimicrobial peptides, which exhibit some degree of selectivity for malignant cells over normal cells. Similar to various clinically approved agents that promote inflammation in the TME, they mediate anticancer effects *in vivo* by promoting tumor infiltration by CTLs and other immune effector cells and depleting immunosuppressive cells (89). Research in various fields has provided newer concepts for the individualized treatment of refractory cancer using cancer vaccines. In addition, toll-like receptor agonists (especially for toll-like receptor 7/8) may provide adjuvant-like signals in combination with vaccines. In MSI cancers, vaccination with frameshift peptide antigens provides a satisfactory and relatively supportive approach. However, the process of antigen recognition is specific to individual tumors, consumes time and resources, and has shown limited effectiveness in improving clinical prognosis.

Adoptive cell therapy can target antigens expressed only on the surface of tumor cells, thus overcoming off-target actions and minimizing side effects. It includes chimeric antigen receptor-T cell therapy, with which adaptive intervention showed remarkably high (approximately 80%) response rates (especially in patients with relapsed or refractory acute lymphoblastic leukemia); this resulted in rapid approval of the agent by the FDA in August 2017 (90). Its potential therapeutic targets include TILs, carcinoembryonic antigen, natural killer group 2D ligands, epidermal growth factor receptor (HER-2), mesothelin, guanylyl cyclase 2C, and mucin 1. Most results are from animal experiments or early clinical trials. Chimeric antigen receptor-T cells constructed with CD19 as the target has shown the most success in the clinic; however, the benefits are limited to B cell-associated hematological tumors. The main challenges of this therapy lie in immunosuppression of the TME and the logistical and cost barriers to treatment.

The bispecific T cell engager (BiTE) is a recombinant bispecific antibody comprising two tandem single-chain variable fragments. It can bind the CD3 antigen on T cells and simultaneously (and uniquely) binds an antigen on tumor cells, thus inducing tumor lysis by the formation of immune synapses between T cells and tumor cells. It passively recruits T cells and redirects endogenous T cells, which kill tumor cells. BiTE therapy (represented by blinatumomab) has achieved remarkable curative effects, mainly in hematologic neoplasms (especially B cell malignancies). Blinatumomab was approved for the treatment of acute lymphocytic leukemia for its high efficacy and safety (91). Its application may be expanded to solid tumors due to the advantages of being MHC-independent, relatively easy-to-produce, T cell receptor-independent, and tumor-infiltrating T cell-independent. The main toxicities of BiTE therapy include cytokine release syndrome and neurotoxicity. Despite the success of BiTE therapy against CD19-expressing B-cell malignancies, a significant number of patients do not respond to the treatment (similar to the case of ICIs); in addition, even if they do respond initially, they eventually relapse. The intrinsic resistance mechanism has not been recognized; present studies are mainly focusing on two aspects, namely, immunosuppressive factors and loss of the CD19 antigen (92).

Oncolytic viruses mainly achieve therapeutic efficacy *via* the dual tumor-killing mechanism of selective tumor cell killing and antitumor immunity. The activation of immunogenic cell death coupled with antigen spread largely contributes to their extended therapeutic effects (93). Their replication in the TME promotes the activation of innate and adaptive immunity for overcoming local immunosuppression and promoting antitumor immunity (94). Regarding its application in gynecological tumors, intraperitoneal oncolytic vaccinia virus (Olvi-Vec) has shown promising safety, clinical activity, and immune activation in patients with platinum-resistant or refractory OC (95).

Intratumoral injection is not the standard treatment for any tumor; nor can it be applied alone. In their study, Sun et al. (32) developed a strategy to reprogram the immunity of the TME by injecting mouse-specific peripheral lymphocyte antigen and IFN-γ intraperitoneally. They confirmed that the combination can activate cytotoxic T cells *via* macrophage-secreted cytokines and antigen presentation, stimulate innate immunity, and also increase the expression of CD69 on CD8+ T cells. The combined efficacy in ovarian tumors of mouse models was found to be significantly higher than that of either agent alone.

## 5 Other types of malignancies

The remarkable success of pembrolizumab and other anti-PD-1 drugs in metastatic CRC inspired its use in advanced EC. In this context, most testing and treatment methods initially developed for CRC are being increasingly used for other tumor types. Therefore, the advancements in immunotherapy for CRC may be potentially applied to gynecologic tumors. Pembrolizumab was approved for first-line treatment of patients with unresectable dMMR/MSI-H metastatic CRC in June 2020. The KEYNOTE-177 study (96) showed that the group receiving pembrolizumab demonstrated significantly longer PFS compared to the chemotherapy group with fewer treatment-related AEs.

The study by Ding et al. (97) was the first to explore the association between resistance to common drugs and fatty acid metabolism-related genes in CRC; the findings suggested an association with ineffectiveness of chemo/immunotherapy, providing a new strategy for predicting curative effects and guiding clinical treatment. The study by Liao et al. (98) evaluated the KRAS-IRF2-CXCL3-CXCR2 axis to identify the biological functions and mechanisms of oncogenic KRASG12D (KRAS*) in ICI resistance; the findings showed that resistance to anti-PD-1 therapy could be overcome in KRAS*-expressing CRC by enforced IRF2 expression or inhibition of CXCR2.

Studies have also sought new potential therapies for a wide range of poor responses to ICI treatment. The study by Ni et al. (99) found that simvastatin (an inhibitor of 3-hydroxy-3-methylglutaryl-coenzyme A reductase) could inhibit PD-L1 expression and promote antitumor immunity by suppressing the expression of long non-coding RNA SNHG29; this indicated its promise as an immunotherapeutic drug. The study by Van den Eynde et al. on the prognosis of metastatic CRC (85) showed that other factors in the multiverse of the TME, such as the Immunoscore, offer superior survival prediction than MSI.

Studies are also evaluating emerging targeted therapy. The study by AbuEid et al. (100) suggested that targeted mitochondrial complex I inhibitors may attenuate melanoma proliferation *in vitro* and progression *in vivo* in mouse melanoma models.

## 6 Discussion

The findings from previous studies suggest that while developing and interpreting predictive biomarkers for cancer immunotherapy, it is necessary to recognize that the acute or chronic activation of certain signaling pathways may have the opposite effect on the immune response against cancer. It is also essential to consider the fact that there may be considerable heterogeneity in individual responses to different forms of immunotherapy. Different variables, such as the variability of host immune systems between individuals and groups, comorbidities, and changes in the TME, may lead to different effects, which warrant further study. Extensive research on the predictors of anti-PD-1 treatment effectiveness and factors related to resistance has shown certain prospects. Studies on these promising factors are not only essential for reducing resistance to immunotherapy, but also for finding promising targets for intervention.

Although it is widely believed that high levels of neoantigen/mutation expression are required for intratumoral T cell accumulation and response to ICIs (especially in patients with LS who predominantly have MSI/dMMR tumors), findings from certain studies challenge this established notion (101).

In addition to the widely studied mechanisms, the newly proposed mechanisms of tumorigenesis provide the rationale for exploring new targets for clinical therapies. Kortlever et al. (102) have explored the obscure internal mechanisms of two oncogenes, namely, KRAS and Myc. These oncogenes cooperate to drive tumorigenesis and co-blockade CCL9 and IL-23; this can markedly inhibit the diverse stromal changes elicited by Myc. Kaymak et al. (103) explored the immune metabolic interactions in the TME and considered it necessary to contextualize the study of immune metabolism. They have therefore provided the basic theoretical network and directions for further targeted research in this field.

In gynecologic cancer, potential markers have been obtained for EC (based on those in CRC); these (especially the MSI/dMMR type) have been verified in many clinical trials (104). Basic experiments on predictive molecules are also being performed in large-scale studies on OC and CC (29). In this context, it is essential to identify biomarkers which may predict ICI efficacy, as it represents a major unmet challenge in the immunotherapy of gynecologic tumors.

In conclusion, further studies are needed to improve and predict clinical responses to immunotherapy and patient outcomes and guide therapeutic decisions in gynecologic cancer. These studies need to focus on composite biomarkers and integrate the composition, localization, and functional orientation of the TME. The findings from this review provide a potentially novel research direction.

## Author contributions

GJ wrote the main manuscript text and prepared the figures and tables. BL provided suggestions on the framework of this review and corrected the mistakes. QW helped with the literature review during writing. All authors contributed to the article and approved the submitted version.
